# Correction to *Lancet Glob Health* 2023; 11: e1075–85

**DOI:** 10.1016/S2214-109X(23)00361-3

**Published:** 2023-07-20

*Asante IA, Hsu SN, Boatemaa L, et al. Repurposing an integrated national influenza platform for genomic surveillance of SARS-CoV-2 in Ghana: a molecular epidemiological analysis.* Lancet Glob Health *2023; **11:** e1075–85*—In this Article, the first sentence of the Findings section of the Summary should have said “(2123 [46·1%] of 4602 samples with complete demographic data were female and 2479 [53·9%] were male)” and the third sentence of the Results section should have said “2123 (46·1%) were female and 2479 (53·9%) were male”. Additionally, in the supplementary appendix, the final cell of row 14 in table S1 should have said “2123 (46·1%)” These corrections have been made as of July 20, 2023.

